# Developmental Trajectories of Size Constancy as Implicitly Examined by Simple Reaction Times

**DOI:** 10.3390/vision5040050

**Published:** 2021-10-19

**Authors:** Irene Sperandio

**Affiliations:** Department of Psychology and Cognitive Science, University of Trento, 38068 Rovereto, Italy; irene.sperandio@unitn.it

**Keywords:** perceived size, retinal size, implicit measure, detection, development

## Abstract

It is still unclear whether size constancy is an innate ability or whether it develops with age. As many developmental studies are limited to the child’s comprehension of the task instructions, here, an implicit measure of perceived size, namely, simple manual reaction time (RT), was opted for based on the assumption that perceptually bigger objects generate faster detection times. We examined size constancy in children (from 5 to 14 years of age) and adults using a simple RT approach. Participants were presented with pictures of tennis balls on a screen that was physically moved to two viewing distances. Visual stimuli were adjusted in physical size in order to subtend the same visual angle across distances, determining two conditions: a small-near tennis ball vs. a big-far tennis ball. Thanks to size constancy, the two tennis balls were perceived as different even though they were of equal size on the retina. Stimuli were also matched in terms of luminance. Participants were asked to react as fast as possible to the onset of the stimuli. The results show that the RTs reflected the perceived rather than the retinal size of the stimuli across the different age groups, such that participants responded faster to stimuli that were perceived as bigger than those perceived as smaller. Hence, these findings are consistent with the idea that size constancy is already present in early childhood, at least from the age of five, and does not require extensive visual learning.

## 1. Introduction

As we watch a train depart from a platform at a railway station, the size of its image on the retina becomes smaller and smaller as the train moves further away from us. Although the train is shrinking on our retina, we tend to perceive it as constant in size and just receding into the distance. This tendency to compensate for distance in computing object size is known as size constancy (for a review, see [1]).

There has been a long-standing debate as to whether size constancy is an innate or a developed ability. On the one hand, a number of studies have supported the notion that size constancy is an innate process. In fact, size constancy has been reported in many species of animals including monkeys (e.g., [2,3]), cats [4], rats [5], ducks [6], dogs [7] and pigeons [8], as well as in early infancy in humans [9,10]. Typically, in these types of studies, where perceptual reports are not an option, head turn responses [11], the direction of reaching movements (e.g., [7]) or the time spent looking at the stimulus, i.e., the habituation paradigm (e.g., [7,10]), are used as a measure of attention to novelty. The rationale behind the habituation paradigm is that an increase in the time that an infant spends looking at an object is related to a perceptual experience of novelty; conversely, a decrease in looking time is related to a perceptual experience of familiarity. Developmental studies on size constancy using this approach have demonstrated that newborn and 4- to 6-month-old infants are able to differentiate between familiar and novel objects even when viewed at different distances, whereby different retinal sizes are generated accordingly (e.g., [10,12,13,14,15]). Specifically, these studies have shown that when infants were not familiarized with the target stimulus, they remained focused on the object placed at varying distances. However, after a familiarization session (habituation), the infants did not follow the target over different distances anymore but shifted their focus to a comparison stimulus, suggesting that they were able to recognize the object regardless of the viewing distance thanks to size constancy. In other words, the target met the expectations of perceived size (i.e., size constancy) and required less attention and looking time than the comparison stimulus, which violated these expectations and attracted the infants’ attention. In fact, without size constancy, the infants would have assumed that the same object placed at different distances would be a novel one due to a change in the retinal image size, thus resulting in an increase in looking time. As such, these findings demonstrate that size constancy is already present in early infancy or even at birth. This implies that size constancy is an organizing principle of perception that does not require visual experience and extensive learning.

On the other hand, however, there are several studies in support of an empiricist view of size constancy according to which this mechanism matures with age. It has been repeatedly shown using size-matching tasks that children up to the age of 9 years tend to exhibit underconstancy, that is, an underestimation of size for distant objects, and that beyond this age, their ability to estimate object size becomes accurate and comparable to adults (e.g., [16,17,18,19,20]). In an attempt to explain this developmental change, two main theories have emerged in the literature: the perceptual learning theory and the metacognitive theory. According to the perceptual learning theory [19,21], the underconstacy observed in early childhood is due to an inability of the developing visual system to process monocular cues to distance, such as texture gradients and linear perceptive cues, which are particularly relevant for an accurate estimation of size when objects are placed at far distances where binocular cues (e.g., vergence and accommodation) are less effective. It is around the age of 9–10 years that the visual system learns how to use monocular cues for the operation of size–distance scaling. According to the metacognitive theory [18,22,23], instead, age-related changes in the accuracy of size estimations of distant objects reported in later childhood are the result of the development of metacognitive awareness of the effects of viewing distance on perceived size. In fact, far objects appear smaller than they actually are. To compensate for this decrease in perceived size with increasing distances, children gradually acquire the ability to deliberately use a cognitive strategy which consists of inflating size estimations of distant objects.

Yet, within the empiricist position, there is still no consensus on the age at which size constancy is fully developed. For example, it has been suggested that basic size constancy mechanisms are present from around the age of five, but that these mechanisms become more efficient once the brain’s ability to process all the cues to distance has developed [24]. Others have proposed that size constancy is fully developed typically by the age of 9, once the visual system has acquired the correct knowledge and application of size–distance principles (e.g., [18]). Moreover, research in support of the metacognitive theory has demonstrated that developmental changes in the use of the distance compensation strategy are observed between 7 and 11 years of age only for far distances and depend on increases in metacognitive awareness and reasoning abilities [18,25]. In contrast, children up to the age of six are unable to use strategies and consistently underestimate object size [17,18] Again, it has been suggested that size constancy abilities reach the final stage of maturation only at the age of 11 [19]. These discrepancies in the literature could be the consequence of methodological issues in measuring perceived size in children.

In research with children, the language used by the experimenter to explain the task is a problematic issue. Many of the studies cited above questioned the participants, ‘which of the stimuli (comparison vs. target) is bigger?’ Participants were then instructed to indicate their choice by pointing or naming the stimulus. However, consideration must be given to the influence that the question type and the level of language understanding can have when measuring perceived size in developmental studies. For example, it has been shown that when words related to size were used as adjectives (e.g., the big cat), 7-year-olds were prone to greater size estimation errors towards the direction of the adjective used, compared to 10-year-olds and adults [26]. Moreover, it is well known that the type of instructions, specifically apparent (i.e., how big the object appears) vs. objective (i.e., how big the object really is) questions, can bias perceptual judgements of size in adults [27,28,29,30,31] as well as in older children [23], such that overconstancy is observed when participants are presented with objective rather than apparent questions. Therefore, when children are explicitly asked to estimate object size, they may generate different attitudes, which may reflect their level of comprehension of the task instructions. This would determine whether object size is judged according to the actual or perceived (apparent) size. The attitude problem is particularly evident in tasks that are heavily based on language, and its impact can be reduced by implementing more indirect tasks.

Interestingly, Sperandio and colleagues [32] introduced a novel approach to investigate size constancy in adults based exclusively on a simple reaction time (RT) task without requiring any explicit reports of size or distance. In this study, pictures of tennis balls were shown at various distances, but scaled in physical size according to distance, in order to generate the same retinal image size. Even though the retinal size of the pictures was kept constant across distances, it was assumed that due to size constancy, the physically bigger and further tennis balls would be perceived as larger than the physically smaller and closer tennis balls. The overall luminance of the stimuli was also controlled for. Participants were asked to press a button as soon as a stimulus appeared on the screen (i.e., detection task). It was found that simple RTs reflected the perceived rather than the retinal size of the stimuli, such that the tennis ball that was physically bigger and placed at the furthest distance was responded to more quickly than the tennis ball that was physically smaller and placed closer to the participant. This RT advantage for perceptually bigger objects was recorded despite the fact that all the stimuli were matched in terms of retinal size and total luminance. A classic finding in psychophysics is that simple RTs are affected by retinal size and luminance in a way that stimuli that subtend greater visual angles or higher luminance produce faster responses [33]. The novelty of Sperandio et al.’s study [32] resides in the fact that simple RTs are also affected by perceived size. This finding suggests that perceived size is an automatic property of object perception that is able to modulate basic behavioral responses, such as detection time. In line with Sperandio et al.’s [32] results, more recent studies have shown that simple RTs are also sensitive to visual illusions: illusory larger objects are responded to more quickly than illusory smaller objects [34,35,36]. The use of such an implicit method in developmental research may allow us to overcome some of the methodical issues discussed above.

The current study aimed to examine whether or not size constancy develops with age using, for the first time, a simple RT approach, as developed by Sperandio et al. [32]. Six different age groups were tested: 5–6, 6–7, 7–8, 9–11 and 12–14 years old and an adult group (>18 years old). The different age ranges were chosen based on when size constancy should be fully established according to the different lines of evidence reviewed above. It was predicted that if size constancy is already present in early childhood, then an RT advantage in response to perceptually bigger objects should be registered across all the age groups. In contrast, if size constancy develops with age, an interaction between age group and size should be expected.

## 2. Materials and Methods

### 2.1. Design

A 2 × 6 mixed design was implemented. There was a within-group factor of physical size, with 2 levels: small and big. There was a between-group factor of age, with 6 levels: 5–6, 6–7, 7–8, 9–11 and 12–14 years old and adults. The dependent variables were accuracy and simple RTs.

### 2.2. Participants

Twenty-six adult participants were recruited via either the University of East Anglia (UEA) Psychology participants’ panel or the UEA SONA system. Participants volunteered for the study and were awarded with either course credits or money for their time. Handedness was assessed using the Edinburgh handedness questionnaire [37].

One hundred and thirty-five child participants were recruited from six different schools in Norfolk (UK). Consent was obtained in writing from the parent/guardian of the child taking part for the youngest age groups (i.e., opt-in consent), whereas an opt-out approach was used for the two older age groups. The final form of consent was established through verbal consent from the child at the beginning of the testing session. After testing, children received a small token gift as compensation for their time. They were asked to report their age in years, along with their gender and handedness. Handedness was confirmed with the observation of the hand used to press the response button during the task. If there was an uncertainty about handedness, the child was additionally asked to draw a picture so that the dominant hand could be determined.

Please refer to Table 1 for demographic information of each age group. Data were only retained from participants who fully completed all the experimental blocks.

It should be noted that the smallest and greatest sample sizes corresponded to the 5–6-year-olds and 9–11-year-olds, respectively. This depended on the availability of participants in the classroom. Testing was conducted according to guidelines and with the approval of the UEA Psychology Ethics review board. All participants (adults and children) had normal or corrected-to-normal vision.

### 2.3. Apparatus and Stimuli

The experiment was created and presented using E-prime version 2.0 software (Psychology Software Tools, Pittsburgh, PA, USA). Visual stimuli were displayed on a Toshiba Tecra PC laptop. The laptop screen was 16 inches in size with a resolution of 1366 × 768 pixels. Responses were collected using a customized USB keyboard, where all buttons but the spacebar had been removed, in order to simplify data collection for the younger participants. Stimuli consisted of big and small images of tennis balls, as used in Sperandio et al.’s study [32]. The luminance of the tennis balls was adjusted using the photo editing software Adobe Photoshop (version 14.2). The background of the screen was set to white (RGBA: 1, 1, 1, 1) and had an average luminance of 98.6 cd/m^2^.

There were two experimental blocks with 60 experimental trials (30 per condition) and 9 catch trials in total. In the ‘big’ block, stimuli consisted of a physically ‘big’ tennis ball, which was 8 cm in diameter with an average luminance of 45 cd/m^2^. In this block, the laptop was always placed 114 cm away from the participant’s eyes. As such, the tennis ball subtended a retinal angle of 4°. In the ‘small’ block, stimuli consisted of a physically ‘small’ tennis ball, which was 4 cm in diameter and had an average luminance of 90 cd/m^2^. In this block, the laptop was always placed at a distance of 57 cm, in order to attain the same retinal image size (4° visual angle) and the same overall luminance of the physically big tennis ball. It should be noted that even if small and big tennis balls generated the same image on the retina, they were perceived as different thanks to size constancy, that is, the physically small and near tennis ball was perceived as smaller than the physically big and far tennis ball.

RTs were recorded in milliseconds (ms) by E-prime and measured the time interval between stimulus onset and button press.

### 2.4. Procedure

Adult participants were tested in the psychophysics laboratory of the School of Psychology at the University of East Anglia, while children were tested in a quiet room at their school. Participants were invited to sit at a table facing the laptop’s screen. The room was dimly lit with a lamp. Participants were instructed to keep their eyes focused on a fixation cross that was presented in the middle of the screen during the entire experiment. The laptop was placed upon a platform to align the screen to the eye level of the participants; however, for the younger and shorter children, the use of the platform was often not necessary. The participant was asked to remain seated in the same position and to avoid movements with their head and body throughout the experiment.

A brief practice session consisting of six trials in total (2 big tennis balls, 2 small tennis balls and 2 catch trials) for the adults and nine trials in total (3 big tennis balls, 3 small tennis balls and 3 catch trials) for the children was used so that participants could familiarize themselves with the task.

The experimental paradigm was adapted from Sperandio et al.’s [32] study. It was shortened in order to be more appealing for younger participants. Each testing session had a maximum duration of 20 min. A standard trial started with a black fixation cross shown in the middle of the screen for 1000 ms. A short warning sound, i.e., a ‘beep’ of 1000 Hz, was then played for 1 s. Next, a random temporal window ranging between 300 and 600 ms preceded the onset of the visual stimulus. The visual stimulus consisted of an image of a tennis ball displayed for 80 ms when older age groups (i.e., 9–11, 12–14, ≥18 years old) were tested or 150 ms when younger age groups (5–6, 6–7, 7–8 years old) were tested. The increased exposure duration of the stimuli for the younger participants was deemed necessary to allow for the additional time required by their developing visual system to process the stimuli. As demonstrated by previous electrophysiological studies, the latency of the early visual components P1 and N1, responsible for the processing of visual stimuli, is longer for children under the age of 9 years old compared to older children and adults [38,39].

**Figure 1:**
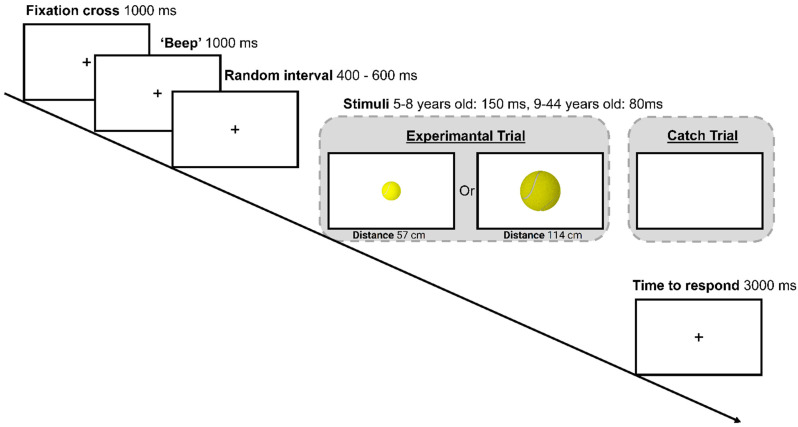
Example of a trial layout. Please note that while only one size of tennis ball was shown in each block, catch trials were used in both blocks. Additionally, the exposure duration of the stimuli was longer in the younger (150 ms) than older (80 ms) age groups.

During the stimulus presentation, participants were asked to press the spacebar as soon as they saw an image of a tennis ball appearing on the screen. To ensure that participants were responding to the visual stimuli and not to the warning auditory cue, in 15% of trials, the tennis ball was replaced by a blank screen, i.e., catch trials, which had the same duration as the experimental stimuli. During the catch trials, participants were instructed to refrain from responding. The following trial started either immediately after the response was made or, if no response was made, after the maximum time limit of 3 s was reached (Figure 1).

Only the child participants were provided with feedback of their performance after each trial. Three types of feedback were pre-recorded and delivered by E-prime: (i) an audible cheer (‘yay’) along with a smiley face for responding to the stimuli within the time limit of 3000 ms and for refraining from responding to catch trials; (ii) a disappointed sound (‘ow’) along with a sad face for slow responses (>3000 ms after stimulus onset); and (iii) an ‘uh-oh’ sound along with a sad face for incorrect responses (i.e., either failing to respond to an experimental trial or responding to a catch trial).

As mentioned above, participants completed two blocks of trials. There was a short break between the two blocks. At the beginning of each block, the experimenter manually moved the laptop to the required location (i.e., ‘near’ for the block of small tennis balls and ‘far’ for the block of big tennis balls), and the participant was reminded to be as quick and as accurate as possible. The presentation order of the two blocks was counterbalanced between participants.

### 2.5. Statistical Analysis

Statistical analyses were performed using the Statistical Package for Social Sciences (SPSS; IBM Corporation; Armonk, NY, USA) version 27. All reported p values are based on two-tailed criteria. A 2 × 6 mixed ANOVA was conducted on both accuracy and RTs with physical size (small vs. big) and age group (5–6 vs. 6–7 vs. 7–8 vs. 9–11 vs. 12–14 vs. ≥18 years old) as the main factors. Partial eta squared (ηp^2^) was calculated to assess effect size. Post hoc tests, corrected for multiple comparisons using the Bonferroni method, were performed to further examine any significant main effect of age group or interaction between physical size and age group. Finally, to account for speed–accuracy trade-offs, a combined measure of performance, namely, the inverse efficiency score (IES; [40]), was calculated for each participant as the means of big and small correct RTs divided by their corresponding percentage accuracies [41]. Therefore, smaller IESs corresponded to faster RTs together with fewer errors.

## 3. Results

### 3.1. Task Accuracy

Accuracy was measured as the percentage of correct responses to experimental and catch trials. A correct response to a catch trial was no response, while a correct response to an experimental trial was an RT between 140 and 650 ms for the adult participants [32]. Therefore, anticipations (RT < 140 ms) and delays (RT > 650 ms) were classified as errors and, as such, removed from the RT analysis. For the child participants, the lower cut-off was the same as that of the adults (140 ms), while an upper cut-off was determined by considering the mean RT +3 SD of the age group. The resulting upper cut-offs were as follows: 1220 ms for the 5–6-year-old and 6–7-year-old groups, 1060 ms for the 7–8-year-old group, 750 ms for the 9–11-year-old group and 710 ms for the 12–14-year-old group.

After removing anticipations, delays and false alarms, several participants were excluded from the analysis due to poor task performance, where accuracy fell below the chance level (50%). Based on this criterion, 24 participants in total were removed from the initial dataset of 156 participants: 7 from the 6–7-year-old group, 2 from the 7–8-year-old group, 9 from the 9–11-year-old group and 6 from the 12–14-year-old group. Two additional participants, one from the 7–8-year-old group and the other from the 9–11-year-old group, were removed from the analysis because they were identified as outliers by Rosner’s test [42]. Therefore, the final sample consisted of 130 participants.

**Figure 2:**
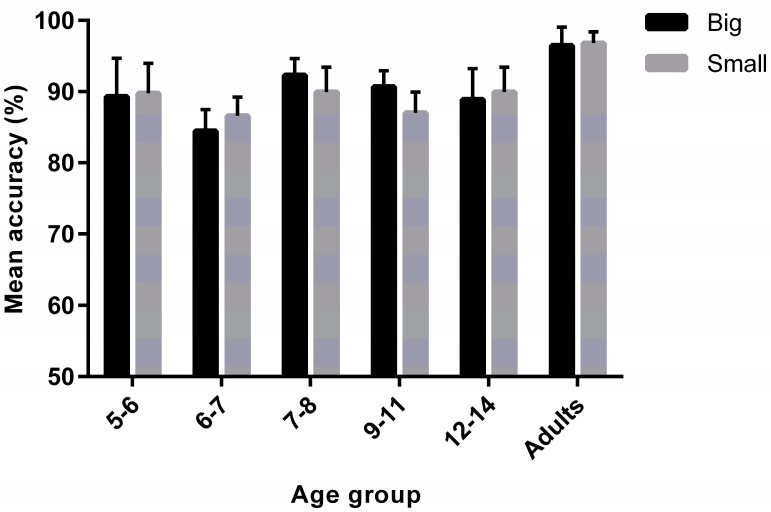
Mean accuracy (%) as a function of physical size (small vs. big) and age group (5–6, 6–7, 7–8, 9–11, 12–14 and ≥18 years old). Error bars represent +/− 95% CIs.

A 2 × 6 mixed ANOVA was conducted on task accuracy with physical size as a within-group factor and age group as a between-group factor. There was no main effect of physical size on task accuracy (F_(1,125)_ = 0.19, *p* = 0.66, ηp^2^ = 0.001). As might be expected [43,44], there was a main effect of age group on task accuracy (F_(5,125)_ = 6.71, *p* < 0.001, ηp^2^ = 0.21), showing that adults were generally more accurate than children. Pairwise comparisons revealed significant differences between adults and all of the other age groups (all p_corr_ ≤ 0.02), aside from the 5–6-year-old (p_corr_ = 0.14) and the 9–11-year-old (p_corr_ = 0.20) groups. All other comparisons did not reach significance (p_corr_ > 0.05). Finally, the interaction between physical size and age group did not reach significance (F_(5,125)_ = 1.79, *p* = 0.12, ηp^2^ = 0.07) (Figure 2).

### 3.2. Simple Reaction Times

RTs were cleaned of anticipations and delays (see above for specific cut-offs). A mixed 2 × 6 ANOVA was conducted on RTs with physical size as a within-group factor and age group as a between-group factor. There was a main effect of physical size on simple RTs (F_(1,125)_ = 6.76, *p* = 0.01, ηp^2^ = 0.05); as previously reported [32], RTs were faster in response to big than small tennis balls (M*_diff_* = −7.44 ms).

**Figure 3:**
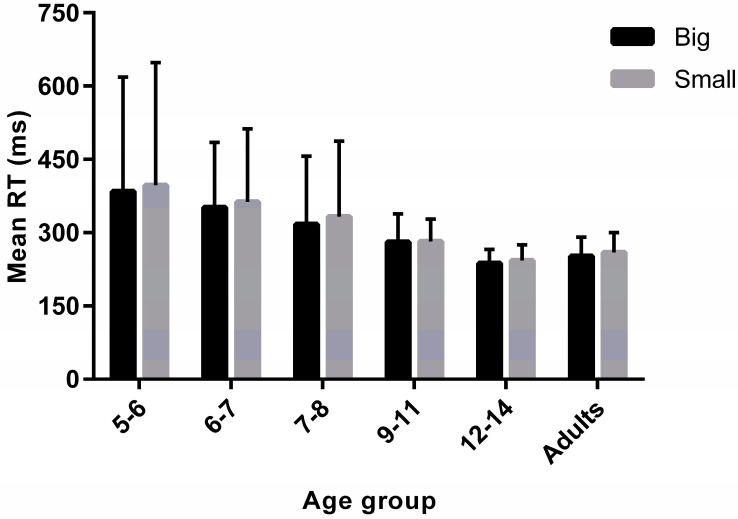
Mean RT (ms) as a function of physical size (small vs. big) and age group (5–6, 6–7, 7–8, 9–11, 12–14 and ≥18 years old). Error bars represent +/− 95% CIs.

As might be expected [43,44], there was also a main effect of age group on simple RTs (F_(5,125)_ = 17.82, *p* < 0.001, ηp^2^ = 0.42). Post hoc comparisons revealed that 5–6-year-olds as well as 6–7-year-olds were significantly slower compared to the older age groups (all comparisons: p_corr_ < 0.001), but not compared to 7–8-year-olds (p_corr_ > 0.05). Moreover, 7–8-year-olds were significantly slower compared to 12–14-year-olds and adults (both comparisons: p_corr_ < 0.01), but not compared to 9–11-year-olds (p_corr_ > 0.05). All other comparisons did not reach significance (p_corr_ > 0.05). Importantly, there was no significant interaction between physical size and age group (F_(5,125)_ = 0.49, *p* = 0.78, ηp^2^ = 0.02), indicating that the RT difference between small and big stimuli did not change as a function of age (Figure 3).

### 3.3. Inverse Efficiency Score

Given that age had an effect on both accuracy and RT, an additional analysis on the IES was carried out to discount possible speed–accuracy trade-offs in the different conditions.

**Figure 4:**
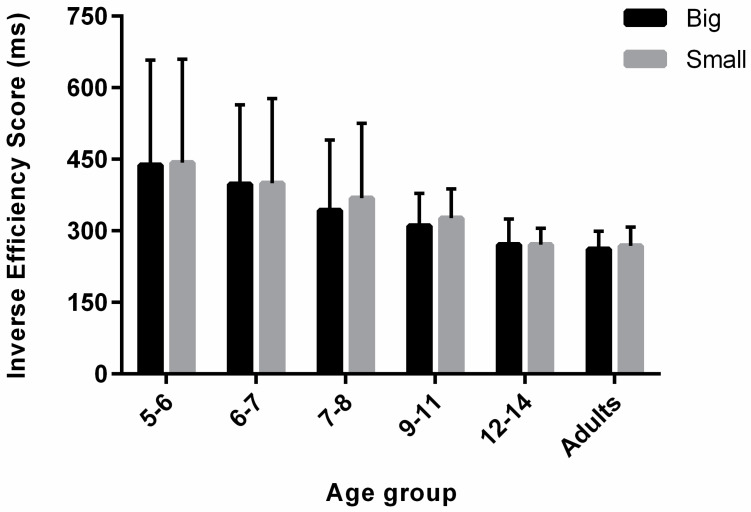
Mean IES (ms) as a function of physical size (small vs. big) and age group (5–6, 6–7, 7–8, 9–11, 12–14 and ≥18 years old). Error bars represent +/− 95% CIs.

A 2 × 6 mixed ANOVA was conducted on the mean IES with physical size as a within-group factor and age group as a between-group factor. There was a main effect of physical size on the IES, with smaller scores (i.e., better performance) for the big stimuli (F_(1,125)_ = 3.99, *p* < 0.05, ηp^2^ = 0.03). There was also a main effect of age group on the IES (F_(5,125)_ = 28.59, *p* < 0.001, ηp^2^ = 0.53). Pairwise comparisons revealed that 5–6-year-olds as well as 6–7-year-olds yielded greater scores compared to the older age groups (all comparisons: p_corr_ ≤ 0.001). Additionally, 7–8-year-olds as well as 9–11-year-olds yielded greater scores compared to the older age groups (all comparisons: p_corr_ ≤ 0.03). All other comparisons did not reach significance (p_corr_ > 0.05). Finally, there was no significant interaction between physical size and age group (F_(5,125)_ = 0.76, *p* = 0.58, ηp^2^ = 0.03) (Figure 4). Despite the lack of interaction, visual inspection of Figure 4 shows that, with respect to the RT analysis in Figure 3, an advantage for bigger objects is now present in the 9–11 years old, whereas it has disappeared for the 12–14 years old, suggesting potential speed–accuracy trade-offs for these age groups.

## 4. Discussion

In the literature, there is still a disagreement as to whether size constancy develops with age and when this mechanism reaches the final stages of maturation. As mentioned in the introduction, this lack of consensus across studies could be related to methodological issues with measuring size perception in children. In particular, the use of explicit measures of perceived size can introduce confounding variables, such as the level of understanding of instructions, general intelligence and demand characteristics. To overcome these issues, here, for the first time, an implicit approach, namely, a simple RT task, was used to investigate the developmental trajectories of size constancy in six age groups: 5–6, 6–7, 7–8, 9–11 and 12–14 years old and adults.

Children’s ability to perform RT tasks has been examined before (e.g., [43,44,45,46,47,48,49]). It is not surprising that children generally exhibit slower RTs than adults. For instance, it has been shown that the mean speed of response of 4-year-olds is around half a second slower than that of adults [44]. Additionally, RTs tend to decrease with an increase in age [44,45,46,47,48,49], and by the age of 15, the speed of response becomes comparable to that of an adult [43]. In keeping with these previous findings, the results show that, overall, children, especially those of the younger age groups, were slower and less accurate than the adults in the simple RT task. In the current study, 5–6-year-olds were, on average, ~140 ms slower than the adults, and the detection time tended to decrease until the age of 12, when the speed of response became comparable to that of an adult. The reduction in RTs observed across the different age groups can be explained by structural changes in the white matter of their developing brains. In fact, white matter growth is critical for an efficient transmission of neural signals, promotes communication between cortical areas and may underlie age-related changes in the processing speed of visual information which are reflected in the RT [50,51,52].

In line with Sperandio et al.’s study [32], it was also observed that RTs followed the rules of size constancy: the detection time was faster in response to perceptually bigger objects than the smaller ones, even if stimuli were matched in retinal size and luminance. This was true across all the tested age groups (except for the 9–11-year-olds (The lack of RT advantage for the big stimuli in 9–11-year-olds is hard to explain. The experimenter who collected the data reported that children of this particular age group appeared, in general, as less motivated and less engaged with the task with respect to the other age groups. Moreover, the IES analysis reported in Figure 4 raises the possibility that a speed–accuracy trade-off might have occurred for this age group. As such, this result should be interpreted with caution)). Such a result provides additional support for the hypothesis that size constancy is already present in early childhood, at least from the age of five. Unfortunately, the present findings cannot reveal if size constancy would operate on RTs in a similar way in children younger than 5 years old. Children below the age of 4 years were excluded from the current study based on the assumption that their RTs would have been unreliable and the task too demanding for them [53]. To overcome this methodological limitation, future studies could use a different implicit measure, namely, visual-evoked potentials (VEPs), to assess size constancy in infancy. Given that a recent ERP study on adults has demonstrated that size–distance scaling emerges in the visually evoked component about 150 ms after stimulus onset [54], it would be interesting to verify if a similar modulation of the ERP signal can be observed in children as well.

In recent years, new results from children suffering from dense bilateral congenital cataracts, who are surgically treated for blindness only years after birth, have furthered the nature–nurture argument on size constancy. Investigating size constancy in this type of population provides the unique opportunity to understand the role of visual experience in size–distance scaling. In two separate studies, it was reported that the newly sighted children were susceptible to two classic visual illusions, namely, the Ponzo illusion and the Müller-Lyer illusion—whose mechanisms are thought to reflect a misapplication of constancy scaling (e.g., [1,55,56])—and that they were able to provide size estimations which adhered relatively well to size constancy rules, immediately after surgery [57,58]. Based on this evidence, one can be tempted to conclude that size constancy does not require extensive visual learning. However, these results should be interpreted with caution. There is still the possibility that these children learned how to use their residual vision (i.e., image blur, light perception, hand movements) as well as the signals originating from other sensory modalities (e.g., haptic, proprioceptive and acoustic inputs) as sources for distance information, allowing for the development of size constancy [58]. This hypothesis is supported by recent findings showing that when healthy participants are asked to perform a size constancy task under restricted viewing conditions, the visual system can benefit from signals that originate from other sensory modalities, such as the somatosensory [59,60,61,62] and auditory [63,64] systems. Hence, these extra-retinal sources of distance information can contribute to size constancy in conditions in which vision does not prevail over other sensory modalities.

The present findings cannot rule out the possibility that some mechanisms of size constancy might be acquired later on in life, such as the ability to use monocular cues to distance [17,18,19,65,66,67]. It should be noted that in the current study, distance was manipulated directly by physically displacing the screen up to 114 cm away from the participants’ eyes. A plethora of evidence indicates that at near viewing distances, such as those tested here, oculomotor adjustments, including vergence and accommodation, are a particularly relevant source of depth information for size constancy [32,59,68,69,70,71,72,73,74,75]—however, see work by Linton who has recently challenged this idea [76,77]. Intriguingly, there is evidence that vergence and accommodative responses start to develop immediately after birth, and that infants become proficient at aligning and focusing both eyes on a target stimulus between two and four months of age [78,79,80,81,82,83,84], highlighting the importance of these signals for the development of visual perception and fine motor coordination in depth. It remains unclear if the same detection profile observed for the different age groups in the current investigation would persist for greater viewing distances, for instance, beyond 12 m, whereby binocular cues become ineffective and distance information is mainly based on monocular cues. A replication of this study with greater viewing distances would also allow us to put the metacognitive theory to the test as age-related changes predicted by this framework should be observed in children aged 7–11 years as a function of the development of their metacognitive awareness and reasoning abilities.

To conclude, this study demonstrates that simple RTs can be recorded in children from the age of five as an implicit measure of perceived size. As such, simple RTs may represent a novel approach for the examination of size constancy in children that could overcome methodological restrictions dictated by the level of language comprehension when assessing perceptual experiences in younger participants.

## Figures and Tables

**Table 1:** Demographic information of participants of each age group.

Age Group	Gender		Handedness			N
	Female	Male	Left	Right	Ambi	
5–6	7	2	2	7	0	9
6–7	11	17	3	25	0	28
7–8	11	8	1	18	0	19
9–11	20	24	6	38	1	44
12–14	14	17	5	26	0	31
18–45	20	5	2	23	0	25
Overall	83	73	19	137	1	156

Note: Age group corresponds to the range of age of the participants.

## Data Availability

The data that support the findings of this study are available from the author upon request.
